# An Advanced Temporal Credential-Based Security Scheme with Mutual Authentication and Key Agreement for Wireless Sensor Networks

**DOI:** 10.3390/s130809589

**Published:** 2013-07-24

**Authors:** Chun-Ta Li, Chi-Yao Weng, Cheng-Chi Lee

**Affiliations:** 1 Department of Information Management, Tainan University of Technology, 529 Zhongzheng Road, Tainan City 71002, Taiwan; E-Mail: th0040@mail.tut.edu.tw; 2 Department of Computer Science, National Tsing Hua University, 101 Kuang-Fu Road, Hsinchu City 30013, Taiwan; E-Mail: cyweng@is.cs.nthu.edu.tw; 3 Department of Library and Information Science, Fu Jen Catholic University, 510 Jhongjheng Road, Sinjhuang Dist., New Taipei City 24205, Taiwan; 4 Department of Photonics and Communication Engineering, Asia University, 500 Lioufeng Road, Taichung City 41354, Taiwan

**Keywords:** cryptanalysis, key agreement, mutual authentication, temporal credential, wireless sensor network

## Abstract

Wireless sensor networks (WSNs) can be quickly and randomly deployed in any harsh and unattended environment and only authorized users are allowed to access reliable sensor nodes in WSNs with the aid of gateways (GWNs). Secure authentication models among the users, the sensor nodes and GWN are important research issues for ensuring communication security and data privacy in WSNs. In 2013, Xue *et al.* proposed a temporal-credential-based mutual authentication and key agreement scheme for WSNs. However, in this paper, we point out that Xue *et al.*'s scheme cannot resist stolen-verifier, insider, off-line password guessing, smart card lost problem and many logged-in users' attacks and these security weaknesses make the scheme inapplicable to practical WSN applications. To tackle these problems, we suggest a simple countermeasure to prevent proposed attacks while the other merits of Xue *et al.*'s authentication scheme are left unchanged.

## 1. Introduction

Wireless sensor networks are innovative *ad hoc* networks that include a large number of sensor nodes with resource-constrained characteristics such as limited power, communication and computational capabilities [1–4]. As soon as sensor nodes are massively and randomly deployed in a target field, the basic functions of the gateway node are to collect sensitive data for authorized users [5,6]. In many cases, a WSN may be deployed in hostile environments and malicious intruders may launch possible attacks for disrupting the normal operations (such as impersonating a legal user to abuse the network resources, inject false messages or invalid sensors into the WSN, launch security attacks and so on) of a WSN. Therefore, entity authentication [7–16] plays an important role in WSNs and logging-in users and deployed sensors should be authenticated to be the admissible participants by the GWN.

In the recent literature, there are a few works that detail a complete secure user authentication schemes for wireless sensor networks with all their different features. In [17] Das proposed an efficient two-factor scheme of user authentication, which is based on easy-to-remember passwords and smart cards. In Das' scheme, it only needs XOR and hashing computations and this reduces the computational complexity, which is suitable for resource-constrained WSNs. Although Das' scheme enhances system performance, it did not make up for the security weaknesses [18–20]. Das' scheme has later attracted a lot of attention and several two-factor user authentication schemes with mutual authentication and key agreement have been proposed in Li *et al.* [20], Yeh *et al.* [21], Das *et al.* [22], Li *et al.* [23], and Xue *et al.* [24]. In [20], Li *et al.* proposed a secure billing service based on the framework of Das' scheme. In [21], Yeh *et al.* introduced an ECC-based user authentication scheme for preventing all the security flaws of the previous scheme [25]. However, in [23], Li *et al.* showed that Yeh *et al.*'s scheme is insecure against several security attacks and further proposed an improved version of Yeh *et al.*'s scheme, which covers all the identified weaknesses and is more efficient for practical WSN environments. In [24], Xue *et al.* suggest a lightweight temporal-credential-based mutual authentication and key agreement scheme that not only provides more functionality features with higher security, but also ensures low costs of computation, communication and storage.

### 1.1. Our Contributions

#### Contributions made in this work can be summarized as follows

- We analyze the security weaknesses of one of the most recent temporal-credential-based authentication schemes for WSNs proposed by Xue *et al.* [24]. Xue *et al.* claimed that their authentication scheme is secure against various known attacks with mutual authentication and key agreement and is suitable for resource-constrained WSNs. However, we find that Xue *et al.*'s authentication scheme still has other security weaknesses such as disclosure of the password and failing to prevent the lost smart card problem and many logged-in users' attacks.
- We propose an advanced scheme to prevent the security threats of Xue *et al.*'s authentication scheme and the phases in our scheme are shown to be efficient in terms of computational complexity and communication overhead.
- Our advanced scheme provides both mutual authentication and key agreement among the user, GWN and the sensor node in wireless sensor networks.
- Our three-party authentication scheme can be used to verify users and sensor nodes without revealing their passwords whenever it is deemed to be necessary.
- A service period feature can be used to revoke users or sensor nodes in a controlled manner and prevent abuse by an authority node GWN.
- Status-bit and login recording features are efficiently implemented and assist in catching misbehaving attackers trying to abuse network resources. The above-mentioned features are especially useful when non-registered attackers attempt illegal activities such as many logged-in user attacks.

### 1.2. Organization of the Paper

The remainder of the paper is organized as follows: Section 2 reviews Xue *et al.*'s authentication scheme [24], whose security weaknesses are shown in Section 3. We propose an advanced authentication scheme with higher security in Section 4, whose security and comparisons of related schemes are analyzed in Section 5 and Section 6, respectively. Section 7 concludes the paper.

## 2. A Review of Xue *et al.*'s Temporal-Credential-Based Authentication Scheme

In this section, we review Xue *et al.*'s temporal-credential-based mutual authentication scheme [24]. This scheme is mainly composed of three phases: registration, login, authentication and key agreement. Moreover, their scheme is composed of three roles: gateway node (GWN), sensor node (*S_j_*) and user (*U_i_*). For convenience of description, we summarize the notations used throughout this paper in Table 1.

### 2.1. Registration Phase

Before registration of the user *U_i_* and the sensor node *S_j_*, each *U_i_* has a secure password pre-shared with GWN and *U_i_*'s identity *ID_i_* and hash value of *U_i_*'s password *H*(*PW_i_*) are stored in GWN's side. Moreover, each *S_j_* has a pre-configured password *PW_i_* and hash value of *S_j_*'s password *H*(*PW_i_*) is stored in GWN's side. This phase has two parts for *U_i_* and *S_j_* and we review them as follows:

(U-1) *U_i_* selects *ID_i_* and computes *VI_i_* = *H*(*TS*_1_‖ *H*(*PW_i_*)) and sends {*ID_i_*, *TS*_1_, *VI_i_*} to GWN via an open and public channel, where *TS*_1_ is current timestamp value of *U_i_*.
(U-2) After receiving the registration request from *U_i_*, GWN checks if |*TS*_1_–T*_GWN_| < Δ*T*, where T*_GWN_ is the current system timestamp of GWN and Δ*T* is the expected time interval for the transmission delay. If it does not hold, GWN sends REJ message back to *U_i_*. Otherwise, GWN retrieves its own copy of *H*(*PW_i_*) by using the key “*ID_i_*”, computes VI_i_* = *H*(*TS*_1_‖ *H*(*PW_i_*)) and checks if VI_i_* = *VI_i_*. If not, GWN terminates it; otherwise, GWN computes *P_i_* = *H*(*ID_i_*‖*TE_i_*), *TC_i_* = *H*(*K_GWN_U_*‖*P_i_*‖*TE_i_*) and *PTC_i_* = *TC_i_*⊕*H*(*PW_i_*) and personalizes the smart card for *U_i_* with the parameters:{*H*(•), *ID_i_*, *H*(*H*(*PW_i_*)), *TE_i_*, *PTC_i_*}.

Before deployment of sensor nodes in a target field, each *S_j_* performs the following steps for registration:

(S-1) *S_j_* computes *VI_j_* = *H*(*TS*_2_‖*H*(*PW_j_*)) and sends {*SID_j_*, *TS*_2_} to GWN via an open and public channel, where *TS*_2_ is current timestamp value of *S_j_*.
(S-2) After receiving the message from *S_j_*, GWN checks if |*TS_2_*–T*_GWN_ | < Δ*T*, where T*_GWN_ is the current system timestamp of GWN and Δ*T* is the expected time interval for the transmission delay. If it does not hold, GWN sends REJ message back to *S_j_*. Otherwise, GWN retrieves its own copy of *H*(*PW_j_*) by using the key “*SID_j_*”, computes VI_j_* = *H*(*TS*_2_‖*H*(*PW_j_*)) and check if VI_j_* = *VI_j_*. If not, GWN terminates it; otherwise, GWN computes *TC_j_* = *H*(*K_GWN_S_*‖ *SID_j_*) and *REG_j_* = *H*(*H*(*PW_j_*)‖*TS*_3_)⊕*TC_j_* and sends {*TS*_3_, *REG_j_*} to *S_j_*.
(S-3) After receiving the message from GWN, *S_j_* checks if |*TS*_3_ – T_j_*| < Δ*T*, where T_j_* is the current timestamp value of *S_j_*. If not, *S_j_* terminates it; otherwise, *S_j_* computes its temporal credential *TC_j_* = *REG_j_*⊕*H*(*H*(*PW_j_*)‖*TS*_3_) and stores it.

### 2.2. Login Phase

If the user *U_i_* wants to access sensor data from the wireless sensor network, *U_i_* inserts a smart card into a terminal and enters *ID_i_* and *PW_i_*. The terminal computes *H*(*H*(*PW_i_*)) and checks the validity of *ID_i_* and *PW_i_* with the stored *ID_i_* and *H*(*H*(*PW_i_*)). If not, the smart card terminates this login request. Otherwise, *U_i_* passes the verification and he/she can read the information stored in the smart card. *U_i_* computes *TC_i_* = *PTC_i_*⊕*H*(*PW_i_*).

### 2.3. Authentication and Key Agreement Phase

(A-1) *U_i_* computes *DID_i_* = *ID_i_*⊕*H*(*TC_i_*‖*TS*_4_), *C_i_* = *H*(*H*(*ID_i_*‖*TS*_4_)⊕*TC_i_*) and *PKS_i_* = *K_i_*⊕*H*(*TC_i_*‖*TS*_4_‖“000”) and sends the mutual authentication message {*DID_i_*, *C_i_*, *PKS_i_*, *TS*_4_, *TE_i_*, *P_i_*} to GWN, where *TS*_4_ is current timestamp value of *U_i_*, *K_i_* is a random key only known to *U_i_* and the binary number “000” is used for distinguishing *H*(*TC_i_*‖*TS*_4_‖“000”) and *H*(*TC_i_*‖*TS*_4_).
(A-2) After receiving the message from *U_i_*, GWN checks the validity of *TS*_4_. If *TS*_4_ is valid for the transmission delay, GWN computes *ID_i_* = *DID_i_*⊕*H*(*H*(*K_GWN_U_*‖*P_i_*‖*TE_i_*)‖*TS*_4_), P_i_* = *H*(*ID_i_*‖*TE_i_*), *TC_i_* = *H*(*K_GWN_U_*‖*P_i_*‖*TE_i_*) and C_i_* = *H*(*H*(ID_i_*‖*TS*_4_)⊕*TC_i_*) and verifies whether C_i_* ≠ *C_i_* or P_i_* ≠ *P_i_*. If it holds, GWN rejects *U_i_*'s login request; otherwise, GWN computes *K_i_* = *PKS_i_*⊕*H*(*TC_i_*‖*TS*_4_‖“000”) and chooses a nearby suitable sensor node *S_j_* as the accessed sensor node. GWN further computes *S_j_*'s temporal credential *TC_j_* = *H*(*K_GWN_S_*‖*SID_j_*), *DID_GWN_* = *ID_i_*⊕*H*(*DID_i_*‖*TC_j_*‖*TS*_5_), *C_GWN_* = *H*(*ID_i_*‖*TC_j_*‖*TS*_5_) and *PKS_GWN_* = *K_i_*⊕*H*(*TC_j_*‖*TS*_5_) and sends {*TS*_5_, *DID_i_*, *DID_GWN_*, *C_GWN_*, *PKS_GWN_*} to *S_j_*, where *TS*_5_ is current timestamp value of GWN.
(A-3) After receiving the message from GWN, *S_j_* checks the validity of *TS*_5_. If *TS*_5_ is valid for the transmission delay, *S_j_* computes *ID_i_* = *DID_GWN_*⊕*H*(*DID_i_*‖*TC_j_*‖*TS*_5_) and $C_{\text{GWN}}^{*}=H\left(ID_{i}\left\|TC_{j}\right\|TS_{5}\right)$ and checks if $C_{\text{GWN}}^{*}=C_{\text{GWN}}$. If not, *S_j_* terminates this session. Else, *S_j_* convinces that the received message is from a legitimate GWN. Moreover, *S_j_* computes *K_i_* = *PKS_GWN_*⊕*H*(*TC_j_*‖*TS*_5_), *C_j_* = *H*(*K_j_*‖*ID_j_*‖*SID_j_*‖*TS*_6_) and *PKS_j_* = *K_j_*⊕*H*(*K_i_*‖*TS*_6_) and sends {*SID_j_*, *TS*_6_, *C_j_*, *PKS_j_*} to *U_i_* and GWN, where *K_j_* is a random key chosen by *S_j_*.
(A-4) After receiving the message from *S_j_*, *U_i_* and GWN separately computes *K_j_*=*PKS_j_*⊕*H*(*K_i_*‖*TS*_6_) and C_j_* = *H*(*K_j_*‖*ID_i_*‖*SID_j_*‖*TS*_6_). For GWN, if C_j_* = *C_j_*, *S_j_* is authenticated by GWN. For the user *U_i_*, if C_j_* = *C_j_*, *S_j_* and GWN are authenticated by *U_i_*. Finally, *U_i_* and *S_j_* can separately compute a common session key *KEY_ij_* = *H*(*K_i_*⊕*K_j_*) and *U_i_* and *S_j_* will use *KEY_ij_* for securing communications in future.

## 3. Security Analysis on Xue *et al.*'s Scheme

Xue *et al.* claimed that their authentication scheme is robust and secure against insider, password guessing and stolen smart card attacks. In fact, based on our security analysis, we observe that Xue *et al.*'s temporal-credential based scheme is insecure against these security requirements. The details of our attacks are as follows.

### 3.1. Stolen Verifier and Insider Attack

In Xue *et al.*'s scheme, GWN needs to maintain the verifier table and it stores each *U_i_*'s identity *ID_i_* and hash value to *U_i_*'s password *H*(*PW_i_*) in GWN's side. In a practical environment, the *PW_i_* chosen by *U_i_* could be short and easily human memorizable, which might be convenient for *U_i_* to remember easily and in practice many users use same identities and passwords to access various online applications or remote servers for their convenience. Thus, we assume that an attacker *U_A_* may steal the password-verifier from GWN's database and launches off-line guessing attacks on it to obtain *U_i_*'s real password *PW_i_*. The details of stolen verifier attack are as follows.

Step 1: *U_A_* steals verifier table from GWN's database and retrieves the hash value of *U_i_*'s password *H*(*PW_i_*).
Step 2: *U_A_* guesses a password PW_i_* and computes *H*(PW_i_*).
Step 3: *U_A_* compares the result of *H*(PW_i_*) with stolen *H*(*PW_i_*).

A match in Step 3 above indicates the correct guessing of *U_i_*'s easy-to-remember password and Xue *et al.*'s authentication scheme then cannot resist the stolen verifier attack. Moreover, if a privileged insider of GWN knows *U_i_*'s password *PW_i_*, he/she may try to use the knowledge of *U_i_*'s *PW_i_* and *ID_i_* to access other applications or servers.

### 3.2. Off-Line Password Guessing Attack

In step (U-1) of registration phase of Xue *et al.*'s scheme, *U_i_* sends{*ID_i_*, *TS*_1_, *VI_i_*} to GWN via an open and public environment, where *TS*_1_ is current timestamp value of *U_i_* and *VI_i_* = *H*(*TS*_1_‖*H*(*PW_i_*)). If an attacker *U_A_* eavesdrops *U_i_*'s registration message {*ID_i_*, *TS*_1_, *VI_i_*}, *U_A_* can launch the off-line password guessing attack by performing the following step:

Step 1: *U_A_* guesses a password PW_i_* and computes VI_i_* = *H*(*TS*_1_‖*H*(PW_i_*)).
Step 2: *U_A_* compares the result of VI_i_* with eavesdropped *VI_i_*.

A match in Step 2 above indicates the correct guessing of *U_i_*'s easy-to-remember password and Xue *et al.*'s authentication scheme suffers from off-line password guessing attack in user side. On the other hand, in step (S-1) of registration phase, *S_j_* sends {*SID_j_*, *TS*_2_, *VI_i_*} to GWN via an open and public environment, where *TS*_2_ is the current timestamp value of *S_j_* and *VI_j_* = *H*(*TS*_2_‖*H*(*PW_j_*)). If an attacker *U_A_* eavesdrops *S_j_*'s registration message {*SID_j_*, *TS*_2_, *VI_j_*}, *U_A_* can launch an off-line password guessing attack by performing the following steps:

Step 1: *U_A_* guesses a password PW_j_* and computes VI_j_* = *H*(*TS*_2_‖*H*(PW_j_*)).
Step2: *U_A_* compares the result of VI_j_* with eavesdropped *VI_j_*.

A match in Step 2 above indicates the correct guessing of *S_j_*'s password and Xue *et al.*'s authentication scheme is then open to an off-line password guessing attack on the sensor side. Moreover, once *U_A_* has successfully guessed *S_j_*'s random password, *U_A_* can use PW_j_* and the eavesdropped message in step (S-2) of the registration phase to derive *S_j_*'s temporal credential *TC_j_* by computing *TC_j_*=*REG_j_*⊕*H*(*H*(PW_j_*)‖*TS*_3_) = *H*(*K_GWN_S_*‖*SID_j_*). Finally, Xue *et al.*'s scheme may suffer from masquerading attacks and an attacker *U_A_* who knows *TC_j_* can easily impersonate the sensor node *S_j_*.

### 3.3. Lost Smart Card Problem

Let us consider the scenario of a lost smart card problem. In the case where *U_i_*'s smart card is lost and it is picked up by an attacker *U_A_*, the stored parameters can be extracted by launching a power analysis attack [22]. As we know, the content of *U_i_*'s smart card is {*H*(•), *ID_i_*, *H*(*H*(*PW_i_*)), *TE_i_*, *PTC_i_*}. With this information, *U_A_* can launch another off-line password guessing attack by performing the following steps:

Step 1: *U_A_* guesses a password PW_i_* and computes *H*(*H*(PW_i_*)).
Step 2: *U_A_* compares the result of *H*(*H*(PW_i_*)) with extracted *H*(*H*(PW_i_*)).

If Step 2 holds, the guessed password PW_i_* is the same as *U_i_*'s real password *PW_i_*. Otherwise, *U_A_* tries another password. Once *U_A_* successfully guesses *U_i_*'s real password, *U_A_* can use PW_i_* and the content of *U_i_*'s smart card to derive *U_i_*'s temporal credential *TC_i_* by computing *TC_i_* = *PTC_i_*⊕*H*(PW_i_*) = *H*(*K_GWN_U_*‖*P_i_*‖*TE_i_*). Thus, Xue *et al.*'s scheme may suffer from masquerading attacks and an attacker *U_A_* who knows *TC_i_* can easily impersonate a legal user *U_i_* to log in to the gateway node and GWN is not aware of having caused any problem.

### 3.4. Many Logged-in Users' Problem

The many logged-in users attack [26,27] means that if a registered user *U_i_*'s smart card is massively duplicated and his/her identity *ID_i_* and password *PW_i_* are exposed to *m* non-registered users *U_a_*, where *a* = 1, 2, …, *m*. Each one who has a smart card and knows *ID_i_* and *PW_i_* can log in to GWN at the same time and GWN is not aware of having caused any problem. In Xue *et al.*'s scheme, each non-registered user *U_a_* generates his/her timestamp *TS_a_* and random key *K_a_* and sends a legal login message {*DID_a_*, *C_a_*, *PKS_a_*, *TS_a_*, *TE_i_*, *P_i_*} to GWN, where *DID_a_* = *ID_i_*⊕*H*(*TC_i_*‖*TS_a_*), *C_a_* = *H*(*H*(*ID_i_*‖*TS_a_*)⊕*TC_i_*) and *PKS_a_* = *K_a_*⊕*H*(*TC_i_*‖*TS_a_*‖“000”). After receiving all the login requests from *U_a_*, GWN gets the same identity *ID_i_* with different timestamps *TS_a_* and random keys *K_a_* and GWN allows them to log in and access *U_i_*'s account simultaneously.

## 4. Advanced Authentication Scheme

In this section, we propose an advanced scheme with strong security. Our advanced scheme consists of four phases, namely pre-registration phase, registration phase, login phase, authentication and key agreement phase. The details of each of these phases are as follows.

### 4.1. Pre-Registration Phase

Before registration of the user *U_i_* and the sensor node *S_j_*, each *U_i_* has a pre-configured pair of identity $ID_{i}^{\text{pre}}$ and password $PW_{i}^{\text{pre}}$ with GWN and the unique parameter $H\left(ID_{i}^{\text{pre}}\|PW_{i}^{\text{pre}}\right)$ and $ID_{i}^{\text{pre}}$ are kept by GWN to check the validity of registration user. Moreover, each *S_j_* has a pre-configured identity *SID_j_* and a 160-bits random number *r_j_* and the hash value of *S_j_*'s pre-configured identity and random number *H*(*SID_j_*‖*r_j_*) and *SID_j_* are stored on the GWN's side.

### 4.2. Registration Phase

**Figure 1.**
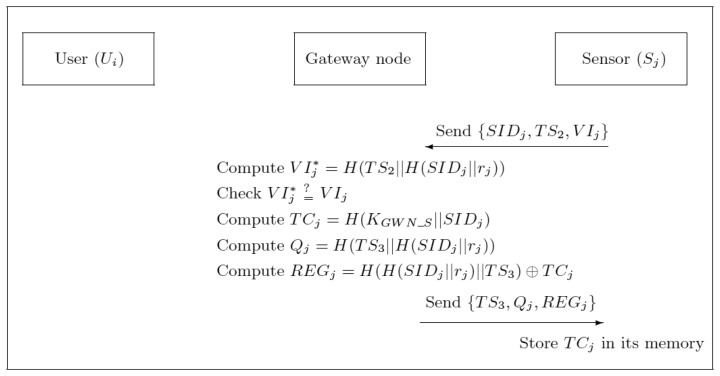
Communication handshakes of the registration phase of the user *U_i_*.

This phase has two parts for *U_i_* and *S_j_* and the details will be described as follows:

(U-1) *U_i_* selects his/her own *ID_i_* and password *PW_i_*. Then *U_i_* computes $VI_{i}=H\left(TS_{1}\|H\left(ID_{i}^{\text{pre}}\|PW_{i}^{\text{pre}}\right)\right)$, $CI_{i}=H\left(ID_{i}^{\text{pre}}\|PW_{i}^{\text{pre}}\right)⊕H\left(ID_{i}\left\|PW_{i}\right\|r_{i}\right)$, $DI_{i}=ID_{i}⊕H\left(ID_{i}^{\text{pre}}\|PW_{i}^{\text{pre}}\right)$ and sends { $ID_{i}^{\text{pre}}$, *TS*_1_, *VI_i_*, *CI_i_*, *DI_i_*} to GWN via an open and public channel, where *TS*_1_ is current timestamp value of *U_i_* and *r_i_* is a random number generated by *U_i_*.
(U-2) After receiving the registration request from *U_i_*, GWN checks if |*TS*_1_–T*_GWN_ | < Δ*T*, where T*_GWN_ is the current system timestamp of GWN and Δ*T* is the expected time interval for the transmission delay. If it does not hold, GWN sends REJ message back to *U_i_*. Otherwise, GWN retrieves its own copy of $H\left(ID_{i}^{\text{pre}}\|PW_{i}^{\text{pre}}\right)$ by using the parameter “ $ID_{i}^{\text{pre}}$”, computes $VI_{i}^{*}=H\left(TS_{1}\|H\left(ID_{i}^{\text{pre}}\|PW_{i}^{\text{pre}}\right)\right)$ and checks if VI_i_* = *VI_i_*. If not, GWN terminates it; otherwise, GWN computes *Q_i_*=*CI_i_*⊕*H*(*ID_i_^pre^*‖*PW_i_^pre^*) = *H*(*ID_i_*‖*PW_i_*‖*r_i_*), *ID_i_* = *DI_i_*⊕*H*(*ID_i_^pre^*‖*PW_i_^pre^*), *P_i_* = *H*(*ID_i_*‖*TE_i_*), *TC_i_* = *H*(*K_GWN_U_*‖*P_i_*‖*TE_i_*) and *PTC_i_* = *TC_i_*⊕*Q_i_* and personalizes the smart card for *U_i_* with the parameters:{*H*(•), *H*(*Q_i_*), *TE_i_*, *PTC_i_*}. Note that GWN maintains a write protected file as depicted in Table 2, where the *Status-bit* indicates the status of the user, *i.e.*, when *U_i_* is logged-in to GWN, the status-bit is set to one, otherwise it is set to zero. Finally, GWN sends *H*(*Q_i_*) and smart card to *U_i_* via an public and open environment.
(U-3) After receiving *H*(*Q_i_*) and smart card from GWN, *U_i_* checks whether the computed *H*(*H*(*ID_i_*‖*PW_i_*‖*r_i_*)) is equal to *H*(*Q_i_*). If they are not equal, *U_i_* aborts this session and the smart card. Otherwise, GWN is authenticated by *U_i_*. *U_i_* enters *r_i_* into his/her smart card and *U_i_*'s smart card contains {*H*(•), *H*(*Q_i_*), *TE_i_*, *PTC_i_*, *r_i_*}. Note that *U_i_* does not need to remember *r_i_* after finishing this phase. The communication handshakes of the registration phase of the user *U_i_* are depicted in Figure 1.

**Figure 2.**
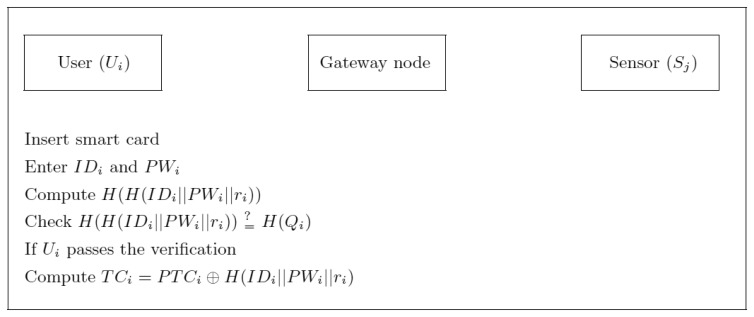
Communication handshakes of the registration phase of sensor node *S_j_*.

Before deployment of sensor nodes in a target field, each *S_j_* performs the following steps for registration.

(S-1) *S_j_* computes *VI_j_* = *H*(*TS*_2_‖*H*(*SID_j_*‖*r_j_*)) and sends {*SID_j_*, *TS*_2_, *VI_j_*} to GWN via an open and public channel, where *TS*_2_ is current timestamp value of *S_j_*.
(S-2) After receiving the message from *S_j_*, GWN checks if |*TS*_2_–T*_GWN_ | < Δ*T*, where T*_GWN_ is the current system timestamp of GWN and Δ*T* is the expected time interval for the transmission delay. If it does not hold, GWN sends REJ message back to *S_j_*. Otherwise, GWN retrieves its own copy of *H*(*SID_j_*‖*r_j_*) by using the key “*SID_j_*”, computes VI_j_* = *H*(*TS*_2_‖*H*(*SID_j_*‖*r_j_*)) and checks if VI_j_* = *VI_j_*. If not, GWN terminates it; otherwise, GWN computes *TC_j_* = *H*(*K_GWN_S_*‖*SID_j_*), *Q_j_* = *H*(*TS_3_*‖*H*(*SID_j_*‖*r_j_*)) and *REG_j_* = *H*(*H*(*SID_j_* ‖*r_j_*) ‖*TS*_3_)⊕*TC_j_* and sends {*TS*_3_, *Q_j_*, *REG_j_*} to *S_j_*.
(S-3) After receiving the message from GWN, *S_j_* checks if |*TS*_3_–T_j_*| < Δ*T*, where T_j_* is the current timestamp value of *S_j_*. If not, *S_j_* terminates it. Otherwise, *S_j_* checks whether the computed *H*(*TS*_3_‖*H*(*SID_j_*‖*r_j_*) is equal to *Q_j_*. If they are equal, *S_j_* computes its temporal credential *TC_j_* = *REG_j_*⊕*H*(*H*(*SID_j_*)‖*r_j_*‖*TS*_3_) and stores it. Note that *S_j_* does not need to store *r_j_* after finishing the phase. The communication handshakes of the registration phase of sensor node *S_j_* are depicted in Figure 2.

### 4.3. Login Phase

**Figure 3.**
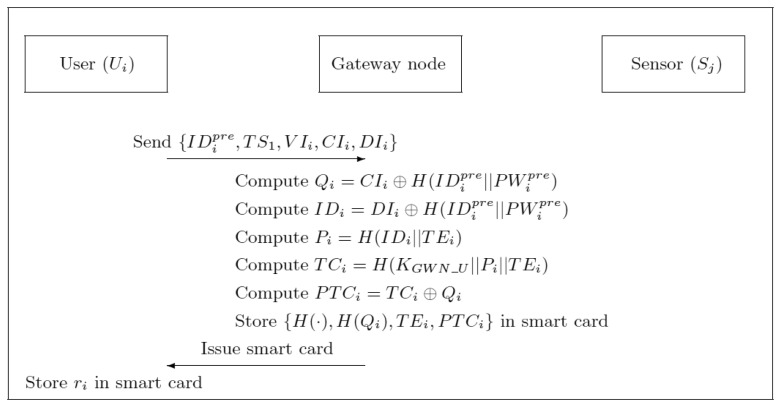
Illustration of the login phase of our advanced scheme.

If the user *U_i_* wants to access sensor data from the wireless sensor network, *U_i_* inserts a smart card into a card reader and enters *ID_i_* and *PW_i_*. The smart card retrieves *r_i_*, computes *H*(*H*(*ID_i_*‖*PW_i_*‖*r_i_*)) ≠ *H*(*Q_i_*), and the smart card terminates this login request. Otherwise, *U_i_* passes the verification and he/she can read the information stored in the smart card. *U_i_* computes *TC_i_* = *PTC_i_*⊕*H*(*ID_i_*‖*PW_i_*‖*r_i_*). The details of the login phase are shown in Figure 3.

### 4.4. Authentication and Key Agreement Phase

(A-1) *U_i_* computes *DID_i_* = *ID_i_*⊕*H*(*TC_i_*‖*TS*_4_), *C_i_* = *H*(*H*(*ID_i_*‖*PW_i_*‖*r_i_*)‖*TS*_4_)⊕*TC_i_*) and *PKS_i_* = *K_i_*⊕*H*(*TC_i_*‖*TS*_4_‖“000”) and *H*(*TC_i_*‖*TS*_4_).
(A-2) After receiving the message from *U_i_*, GWN checks the validity of *TS*_4_. If *TS*_4_ is valid for the transmission delay, GWN computes TC_i_* = *H*(*K_GWN_U_*‖*P_i_*‖*TE_i_*) and *ID_i_* = *DID_i_*⊕*H*(TC_i_*‖*TS*_4_) and retrieves *U_i_*'s password-verifier of *Q_i_* = *H*(*ID_i_*‖*PW_i_*‖*r_i_*) by using the parameter “*ID_i_*”. Then, GWN further computes C_i_* = *H*(*H*(*Q_i_*‖*TS*_4_)⊕*TC_i_*) and verifies whether C_i_* = *C_i_*. If it does not hold, GWN rejects *U_i_*'s login request; otherwise, the status-bit is set to one and *TS*_4_ is recorded in the 4th field of the identity table to demonstrate *U_i_*'s last login. GWN computes *K_i_* = *PKS_i_*⊕*H*(*TC_i_*‖*TS*_4_‖“000”) and chooses a nearby suitable sensor node *S_j_* as the accessed sensor node. GWN further computes *S_j_*'s temporal credential *TC_j_* = *H*(*K_GWN_S_*‖*SID_j_*), *DID_GWN_* = *ID_i_*⊕*H*(*DID_i_*‖*TC_j_*‖*TS*_5_), *C_GWN_* = *H*(*ID_i_*‖*TC_i_*‖*TS*_5_) and *PKS_GWN_* = *K_i_*⊕*H*(*TC_j_*‖*TS*_5_) and sends {*TS*_5_, *DID_i_*, *DID_GWN_*, *C_GWN_*, *PKS_GWN_*} to *S_j_*, where *TS*_5_ is current timestamp value of GWN.
(A-3) After receiving the message from GWN, *S_j_* checks the validity of *TS*_5_. If *TS*_5_ is valid for the transmission delay, *S_j_* computes *ID_i_* = *DID_GWN_*⊕*H*(*DID_i_*‖*TC_j_*‖*TS*_5_) and C*_GWN_ = *H*(*ID_i_*‖*TC_j_*‖*TS*_5_) and check if C*_GWN_ = *C_GWN_*. If not, *S_j_* terminates this session. Else, *S_j_* convinces that the received message is from a legitimate GWN. Moreover, *S_j_* computes *K_i_* = *PKS_GWN_*⊕*H*(*TC_j_*‖*TS*_5_), *C_j_* = *H*(*K_j_*‖*ID_i_*‖*SID_i_*‖*TS*_6_) and *PKS_j_* = *K_j_*⊕*H*(*K_i_*‖*TS*_6_) and sends{*SID_j_*, *TS*_6_, *C_j_*, *PKS_j_*} to *U_i_* and GWN.
(A-4) After receiving the message from *S_j_*, *U_i_* and GWN separately computes *K_j_* = *PKS_j_*⊕*H*(*K_i_*‖*TS*_6_) and C_j_* = *H*(*K_j_*‖*ID_i_*‖*SID_j_*‖*TS*_6_). For GWN, if C_j_* = *C_j_*, *S_j_* is authenticated by GWN. For the user *U_i_*, if C_j_* = *C_j_*, *S_j_* and GWN are authenticated by *U_i_*. Finally, *U_i_* and *S_j_* can separately compute a common session key *KEY_ij_* = *H*(*K_i_*⊕*K_j_*) and *U_i_* and *S_j_* will use *KEY_ij_* for securing communications in future.

**Figure 4.**
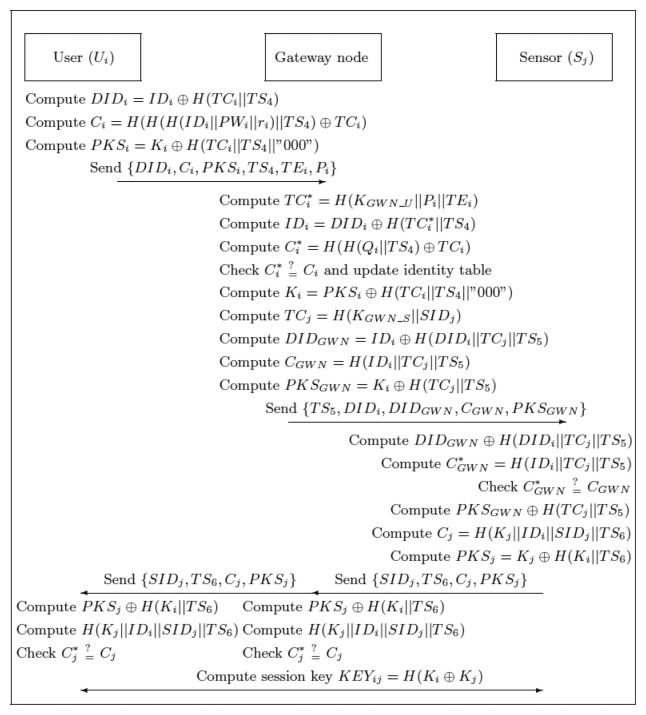
Illustration of the authentication and key agreement phase of our advanced scheme.

After finishing the authentication and key agreement phase, the identity table is updated and the content of the identity table is shown in Table 3. The detailed steps of the authentication and key agreement phase are shown in Figure 4.

## 5. Security Analysis on Our Advanced Authentication Scheme

In this section, for security analysis on our advanced authentication scheme, we use the threat model described in Section 3 and show that our proposed scheme can withstand the following security attacks. Let us consider the following threat scenarios.

–Scenario 1 We assume that a privileged-insider of GWN can steal *U*_i_'s identity and password verifier from the GWN's identity table.
– Scenario 2 We assume that an attacker can eavesdrop *U*_i_'s registration message.
– Scenario 3 We assume that a legal user's smart card has been stolen or lost and the attacker can extract the secret parameters stored in the smart card.
– Scenario 4 We assume that *U_i_*'s identity *ID_i_*, password *PW_i_* and login parameters {*H*(•), *H*(*Q_i_*), *TE_i_*, *PTC_i_*, *r_i_*} are leaked to more than one non-registered users.

### 5.1. Resistance to Stolen Verifier and Insider Attacks

In registration phase of our advanced authentication scheme, *U_i_* registers to GWN by presenting *Q_i_* = *H*(*ID_i_*‖*PW_i_*‖*r_i_*) instead of *PW_i_* and *H*(*PW_i_*). For the threat model in Scenario 1, we assume that a privileged-insider of GWN can steal *U*_i_'s identity and password-verifier from GWN's identity table. Note that the value of *r_i_* is not revealed to GWN and the bit length of |*r_i_*| is large enough. If SHA-256 is used in our advanced scheme, the attacker may attempt to derive *PW_i_* and *r_i_* from password-verifier *Q_i_* = *H*(*ID_i_*‖*PW_i_*‖*r_i_*). Due to the intractability under the assumption of a secure one-way hashing function and the bit-length of *r_i_* is 160 bits. Thus, the probability to guess correct *r_i_* is *1/2^160^*. Moreover, the attacker must guess a correct password *PW_i_* and the probability to guess a correct *p* character *PW_i_* approximated to *1/2^6p^*. Therefore, it is computationally infeasible for the attacker to derive *U_i_*'s password *PW_i_* and random number *r_i_* at the same time because the probability approximated to *1/2^(6p^*^+^*^160)^*. As a result, a privileged-insider still cannot derive *U_i_*'s real password *PW_i_* by performing off-line password guessing attacks on *H*(*ID_i_*‖*PW_i_*‖*r_i_*) and our advanced authentication scheme is secure against stolen verifier and insider attacks.

### 5.2. Resistance to Off-Line Password Guessing Attacks

In step (U-1) of registration phase of our scheme, *U_i_* sends { $ID_{i}^{\text{pre}}$, *TS*_1_, *VI_i_*, *CI_i_*, *DI_i_*} to GWN via an open and public environment. For the threat model in Scenario 2, if an attacker *U_A_* eavesdrops *U_i_*'s registration message { $ID_{i}^{\text{pre}}$, *TS*_1_, *VI_i_*, *CI_i_*, *DI_i_*}. First, *U_A_* cannot derive *U_i_*'s password-verifier *H*(*ID_i_*‖*PW_i_*‖*r_i_*) from $CI_{i}=H\left(ID_{i}^{\text{pre}}\|PW_{i}^{\text{pre}}\right)⊕H\left(ID_{i}\left\|PW_{i}\right\|r_{i}\right)$ because *U_A_* does not know *U_i_*'s unique parameter $H\left(ID_{i}^{\text{pre}}\|PW_{i}^{\text{pre}}\right)$. Second, *U_i_*'s password-verifier *H*(*ID_i_*‖*PW_i_*‖*r_i_*) is under protection of a one-way hashing function and it is computationally infeasible without knowing *U_i_*'s identity *ID_i_*, password *PW_i_* and the random number *r_i_*. We assume the bit-length of *ID_i_* is *q* characters and the probability to guess a correct *m* character *ID_i_* approximated to *1/2^6q^*. Therefore, it is computationally infeasible for the attacker to derive *U_i_*'s identity *ID_i_*, password *PW_i_* and random number *r_i_* at the same time because the probability approximated to *1/2^(6p^*^+^*^6q^*^+^*^160)^*. On the other hand, in step (S-1) of registration phase of our scheme, *S_j_* registers to GWN by presenting {*SID_j_*, *TS*_2_, *VI_j_* = *H*(*TS*_2_‖*H*(*SID_j_*‖*r_j_*))} instead of *PW_j_* and *H*(*PW_j_*). Therefore the attacker cannot launch an off-line guessing attack unless he/she knows the random number *r_j_*. In this case, a possible off-line password guessing attack on user or sensor side is not working in our advanced scheme.

### 5.3. Resistance to Smart Card Lost Problem

The smart card lost problem is an inherent limitation of remote user authentication schemes. For the threat model in Scenario 3, we assume that *U_i_*'s smart card has been stolen or lost and the attacker *U_A_* can extract the secret parameters {*H*(•), *H*(*Q_i_*), *TE_i_*, *PTC_i_*, *r_i_*} stored in the smart card. However, in order to log in to GWN by using *U_i_*'s lost or stolen smart card, *U_A_* needs to guess real identity *ID_i_* and password *PW_i_* correctly at the same time. In fact, it is computationally infeasible to guess these two parameters correctly at the same time in polynomial time since *ID_i_* and *PW_i_* are well-protected by a one-way hashing function. Therefore, our proposed scheme can withstand this type of attack too.

### 5.4. Resistance to the Many Logged-in Users Problem

For the threat model in Scenario 4, we assume that *U_i_*'s identity *ID_i_*, password *PW_i_* and parameters {*H*(•), *H*(*Q_i_*), *TE_i_*, *PTC_i_*, *r_i_*} are leaked to more than one non-registered users. However, the gateway node GWN maintained a status-bit field and a last login field in its identity table. Therefore, no one is allowed to login GWN at the same time out of all who know *ID_i_*, *PW_i_* and valid parameters {*H*(•), *H*(*Q_i_*), *TE_i_*, *PTC_i_*, *r_i_*}. Based on the protection of GWN's identity table, the advanced scheme is secure against many logged-in users attacks.

## 6. Comparisons of Related Schemes

In this section, we will analyse the functionality and performance of our advanced scheme and compare it with Xue *et al.*'s scheme [24] and other related schemes [17,21]. Functionality and performance comparisons of our scheme and other related schemes [17,21,24] are shown in Table 4 and Table 5, respectively. In Table 4, we can see that our advanced scheme not only provides proper password protection and secure service billing, but also prevents many logged-in users attack and other attacks. According to the analysis results reported in [10,24], the time complexity of various operations in terms of *T_H_* and *T_ECC_* are listed in Table 5. We have compared the computational complexity using both formulated results and rough quantitative analysis in Table 5 for different phases: the registration, login and authentication phases of [17,21,24], and our scheme. For example in the test environment (CPU: 2.4 GHz, RAM: 4.0 G), we have run it 100 times to get the average result. *T_H_* is about 3,000 times faster than *T_ECC_* (*T_H_* is nearly 0.0002 second on average when using SHA-256 and *T_ECC_* is nearly 0.6 second on average when using ECC-160). Our advanced scheme, Yeh *et al.* [21] and Xue *et al.* [24] all provide the functions of session key agreement and mutual authentication between each two of the user, GWN and the sensor node.

Moreover, our scheme and Xue *et al.* [24] both provide the service billing function. Our advanced scheme requires 9*T_H_* for the user, 6*T_H_* for the sensor node and 11*T_H_* for GWN. Assume *T_H_* = 0.0002 second and *T_ECC_* = 0.6 second according to our simulation.

Compared with other three schemes which cannot ensure password protection, all participants in three phases of our advanced scheme require about 0.0052 seconds, which can be almost ignored, so our advanced scheme does not increase too much computational complexity while providing more function requirements and preventing more security attacks.

## 7. Conclusions

In this paper, we have analyzed the vulnerability and security attacks existing in Xue *et al.*'s temporal-credential-based mutual authentication scheme and proposed an advanced secure authentication scheme which can satisfy mutual authentication and key agreement between the user, the gateway node and the sensor node. Compared to the existing schemes, our advanced scheme supports extra functionalities such as user password protection and login recording strategy for enhancing the system security. In addition, through the use of lightweight one-way hashing computation, our authentication scheme significantly reduces the implementation cost. Through informal security analysis, we have shown that our proposed scheme has the ability to resist various known attacks, including stolen verifier attacks, insider attacks, lost smart card problems and many logged-in users attack, *etc.* As a result, extra functionalities are added and its higher security along with low computational cost make our advanced scheme very appropriate for securing wireless sensor networks in practice.

## Figures and Tables

**Table 1.** Notations used throughout this paper.

**Symbol**	**Description**
*U_i_*	User
*S_j_*	Sensor node
GWN	Gateway node
*ID_i_/PW_i_*	Identity/Password of the user *U_i_*
*SID_j_/PW_j_*	Pre-configured identity/password of the sensor node *S_j_*
*K_GWN_U_*/*K_GWN_S_*	Two private system parameters only know to GWN
*TC_i_/TC_j_*	A temporal credential issued by GWN to *U_i_*/*S_j_*
*TS*	The timestamp value
*KEY_ij_*	The shared session key between *U_i_* and *S_j_*
*TE_i_*	The expiration time of *U_i_*'s temporal credential
⊕	The bitwise exclusive-OR operation
*H*(•)	The one-way hashing function
‖	The bitwise concatenation operation

**Table 2.** The identity table of GWN after finishing the registration phase.

**User Identity**	**Password-Verifier**	**Status-Bit**	**Last Login**	**Service Period**
…	…	…	…	…
*ID_i_*	*Q_i_*	0/1	N/A	*TE_i_*
…	…	…	…	…

**Table 3.** The identity table of GWN after finishing the authentication and key agreement phase.

**User Identity**	**Password-Verifier**	**Status-Bit**	**Last Login**	**Service Period**
…	…	…	…	…
*ID_i_*	*Q_i_*	0/1	*TS*_4_	*TE_i_*
…	…	…	…	…

**Table 4.** Functionality comparisons of our advanced scheme and related schemes.

**Items/Schemes**	**Das [17] (2009)**	**Yeh *et al.*[21] (2011)**	**Xue *et al.*[24] (2013)**	**Our Advanced Scheme**
Mutual authentication	No	Yes	Yes	Yes
Key agreement	No	Yes	Yes	Yes
Password protection	No	No	No	Yes
Provision of service billing	No	No	Yes	Yes
Resistant to stolen verifier attack	Yes	Yes	No	Yes
Resistant to insider attack	No	Yes	No	Yes
Resistant to lost smart card attack	No	No	No	Yes
Resistant to many logged-in users' attack	No	No	No	Yes

**Table 5.** Performance comparisons of our advanced scheme and related schemes.

**Participant/Computations**	**Das [17] (2009)**	**Yeh *et al.*[21] (2011)**	**Xue *et al.*[24] (2013)**	**Our Advanced Scheme**
User (*U_i_*)	4 *T_H_*	1 *T_H_* + 2 *T_ECC_*	7 *T_H_*	9 *T_H_*
Sensor (*S_j_*)	1 *T_H_*	3 *T_H_* + 2 *T_ECC_*	5 *T_H_*	6 *T_H_*
Gateway node (GWN)	7 *T_H_*	4 *T_H_* + 4 *T_ECC_*	10 *T_H_*	11 *T_H_*
Computation costs	12 *T_H_*	8 *T_H_* + 8 *T_ECC_*	22 *T_H_*	26 *T_H_*
Computation time	0.0024 s	4.8016 s	0.0044 s	0.0052 s

*T_H_*: Time for SHA-256 one-way hashing computation; *T_ECC_*: Time for ECC-160 encryption/decryption computation; s: Second.
